# 3D Printing of Polyacrylamide/Sodium Alginate/Ammonium Molybdate/Lithium Chloride Hydrogels for E-Skin and Information Encryption

**DOI:** 10.3390/gels11090703

**Published:** 2025-09-02

**Authors:** Qinglin Wang, Yinghao Zhao, Hao Zeng, Xiaohu Chen, Chunliang Chen, Jiashu Cui, Yanen Wang

**Affiliations:** 1 Department of Industry and Engineering, School of Mechanical Engineering, Northwestern Polytechnical University, Xi’an 710072, China; wql0424@mail.nwpu.edu.cn (Q.W.); zhaoyinghao@mail.nwpu.edu.cn (Y.Z.); haozeng@mail.nwpu.edu.cn (H.Z.); chenxh@mail.nwpu.edu.cn (X.C.); ccl@mail.nwpu.edu.cn (C.C.); cuijs@mail.nwpu.edu.cn (J.C.); 2 Bio-Additive Manufacturing University-Enterprise Joint Research Center of Shaanxi Province, Northwestern Polytechnical University, Xi’an 710072, China

**Keywords:** photochromic hydrogel, 3D printing, information encryption, electronic skin

## Abstract

With the rapid development of flexible electronic skin materials, the demand for ion-conductive hydrogels is constantly growing. Specifically, these ion-conductive hydrogels are required to simultaneously exhibit excellent mechanical properties, high conductivity, and multifunctionality. Moreover, this performance requirement needs to be met in complex environments. However, the rapid production of hydrogels that combine high conductivity and photochromic properties remains a major challenge. In this study, a simple one-pot method was employed to successfully prepare multifunctional photochromic hydrogels by incorporating ammonium molybdate (Mo_7_) and lithium chloride (LiCl) into a dual-network hydrogel composed of polyacrylamide (PAAm) and sodium alginate (SA). PAAm/SA/Mo_7_/LiCl (PSML) hydrogels exhibit excellent comprehensive performance, including superior conductivity (average value of 164 S/cm), rapid UV response time (<20 s), good color-changing reversibility, outstanding high stretchability (peak value of 2800%), and high transparency (>70%). The design ingeniously combines two types of synergistic effects: the synergistic effect of the dual-network structure and that of the multifunctional component functional additives (Mo_7_, LiCl). Specifically, the PSML hydrogel integrates photochromic properties, excellent mechanical properties, good anti-freezing properties, and 3D printability through this design. Due to these outstanding properties, the PSML hydrogel shows broad application prospects in fields such as flexible strain sensors, information storage, and encryption devices.

## 1. Introduction

Flexible electronic skin (e-skin) has emerged as a research hotspot in recent years [1,2,3], demonstrating vast potential in human–machine interaction [4], health monitoring [5,6], wearable devices [2,7,8], and smart sensing [9,10]. Ionic hydrogels are considered to be ideal materials for constructing flexible electronic skin due to their high conductivity, mechanical adjustability, and biocompatibility [11,12,13,14]. Wearable applications require ion-conductive hydrogels to have excellent mechanical properties and frost resistance to cope with complex environments [15,16]. More importantly, they also need to have intelligent properties such as optical response and customization to meet the multifunctional needs of smart wearable devices [17,18,19]. Although the development of multifunctional ion-conductive hydrogels has become a new trend, how to achieve this remains a major challenge [18,20].

Photochromic materials have garnered significant attention in the fields of information display and anti-counterfeiting due to their striking color contrast and information visualization capabilities [21,22,23]. These materials can be categorized into two types based on their chemical composition: inorganic and organic compounds. Compared to organic compounds, inorganic photochromic materials offer advantages such as faster color-changing rates and higher thermal stability [24,25]. Ammonium molybdate (Mo_7_) is a typical inorganic photochromic material that combines excellent biocompatibility with a stable chemical structure [26,27]. When exposed to ultraviolet light, Mo_7_ rapidly transitions from colorless to blue-green [28,29]. Based on these properties, Mo_7_ is commonly used as a low-cost color-changing medium in information display and encryption applications [30,31,32]. For example, Xue et al. [17] encapsulated Mo_7_ and glycerol in a hydrogel using a semi-interpenetrating polymer network (semi-IPN) made from polyacrylamide (PAAm) and hydroxypropyl cellulose (HPC), synthesizing a multifunctional polyformaldehyde-based hydrogel. This hydrogel exhibits high conductivity (0.142 S/cm), rapid UV response time (20 s), high stretchability (606%), and reversible color change. Through a patterned mask method, the hydrogel was validated for application in information storage devices and ink-free printing fields. Zhou et al. [33] developed a composite hydrogel with tunable visual and electrical properties by incorporating (NH_4_)_6_Mo_7_O_24_ nanoparticles (Mo_7_ NPs) and calcium alginate (Ca-Alg) ion supramolecular networks into a PAAm hydrogel. The hydrogel can generate visual signals when exposed to ultraviolet light and produce modulated digital signals in response to near-infrared light pulses to simulate biological synapse functions. A multi-level information decryption system constructed using hydrogel synapse arrays combines visual information camouflage and digital information decoding functions, which can effectively enhance information security. However, this system cannot be built quickly due to its high complexity. Therefore, how to achieve rapid manufacturing of three-dimensional information encryption structures remains an area requiring further exploration [34,35].

As an emerging manufacturing method, 3D printing technology offers a promising approach for the personalized three-dimensional fabrication of flexible electronic skin due to its high precision and flexible, controllable structure [36]. In particular, photocuring 3D printing technology based on digital light processing (DLP) can efficiently and precisely construct hydrogel devices with complex three-dimensional structures through layer-by-layer photopolymerization. This technology uses photosensitive prepolymers to undergo cross-linking reactions under light of a specific wavelength to achieve rapid molding of hydrogels, with advantages such as high resolution, good molding efficiency, and structural controllability [37,38]. Combining photocuring 3D printing with smart hydrogel materials not only enables personalized customization and rapid manufacturing, but also imbues hydrogel structures with multifunctional properties such as photoresponsiveness, electrical conductivity, or environmental adaptability, supporting their application in cutting-edge applications such as electronic skin, information encryption, and wearable devices [39,40].

In this study, Mo_7_ and LiCl were introduced into the double network hydrogel of PAAm and (sodium alginate) SA using a one-pot method to prepare a PAAm/SA/Mo_7_/LiCl (PSML) photochromic hydrogel with fast response (20 s) and high toughness. The hydrogel combines the brittle network of SA with the flexible network of PAAm, which significantly improves its mechanical properties. In addition, Mo_7_ endows the hydrogel with reversible photochromic properties (stable for 20 cycles). LiCl further improves the conductivity and anti-freezing properties of the hydrogel. Based on its rapid photoresponse (<20 s), a three-dimensional hydrogel with a QR code pattern was innovatively fabricated using 3D printing, providing proof-of-concept for information encryption applications. With these excellent properties, PSML-2 hydrogel is expected to be applied in human health monitoring, optoelectronic displays, information security, and other fields.

## 2. Results and Discussion

### 2.1. Microtopography of Hydrogels

**Figure 1:**
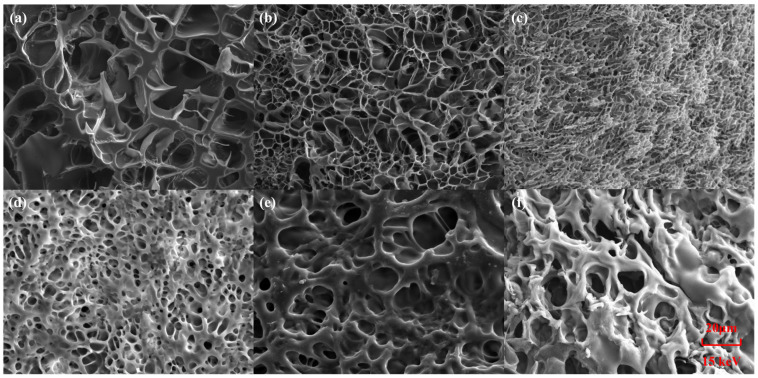
The SEM images (2000×) of (**a**) PAAm; (**b**) PAAm/SA; (**c**) PAAm/SA/Mo_7_; (**d**) PSML-1; (**e**) PSML-2; (**f**) PSML-3 hydrogels.

**Figure 2:**
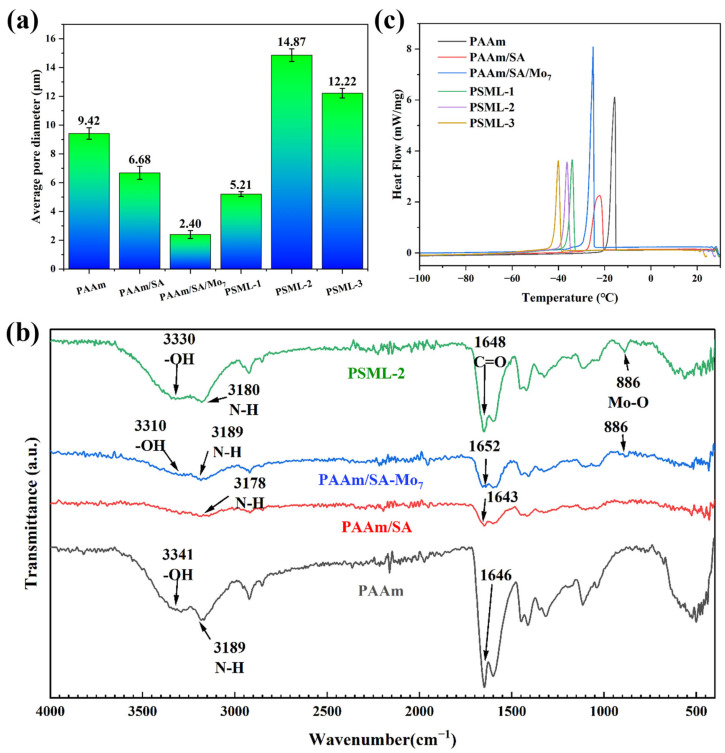
(**a**) Pore size statistics of hydrogels; (**b**) FTIR spectra of PAAm, PAAm/SA, PAAm/SA/Mo_7_, PSML-2 hydrogels; (**c**) DSC curves of hydrogels.

As shown in Figure 1, SEM images of the internal structures of different hydrogels were taken under a tungsten filament scanning electron microscope. The results show that all hydrogels have a three-dimensional porous structure, which provides conditions for the uniform distribution of ion-conducting fillers. The pore size of PSML hydrogels (Figure 1d–f) increases significantly with the increase in the mass fraction of LiCl in the composition, which may further lead to a decrease in the mechanical properties of the hydrogels. The microstructure of the hydrogel was characterized by SEM (Figure 2a). The average pore size, quantified through image analysis of multiple random regions (*n* = 5 pores per sample), is reported. Importantly, the freeze-drying preparation necessary for SEM imaging induces substantial structural shrinkage. Consequently, the observed pore sizes are approximations and are likely much smaller than the actual dimensions of the water-swollen network in its native state.

### 2.2. Chemical Structure of the Hydrogel

As shown in Figure 2b, a series of characteristic peaks of PAAm can be observed. The -OH stretching peak is at 3341 cm^−1^, the N-H antisymmetric vibration peak is at 3189 cm^−1^, and the C=O stretching vibration peak appears at 1646 cm^−1^. PAAm/SA hydrogel forms a double-layered cross-linked network structure upon copolymerization with SA. As a result, the C=O stretching vibration peak shifts to 1643 cm^−1^, the N-H antisymmetric vibration peak shifts to 3178 cm^−1^.

After the addition of PAAm/SA/Mo_7_, PAAm/SA/Mo_7_ exhibited a significant absorption peak at 886 cm^−1^, indicating the presence of Mo-O in the hydrogel [41]. The addition of lithium chloride caused the -OH stretching peak to shift from 3341 cm^−1^ to 3330 cm^−1^, indicating that PSML-2 forms stronger hydrogen bonds through the Hofmeister effect [36] salting out. These strong hydrogen bonds act as additional physical cross-linking points, significantly improving the tensile strength of the material.

### 2.3. Anti-Freezing Properties of Hydrogels

The anti-freezing properties of PSML hydrogel were investigated using the DSC method (Figure 2c). PAAm and PAAm/SA hydrogels showed endothermic peaks at −15 °C and −20 °C. The addition of Mo_7_ and LiCI allowed PSML-3 hydrogels to remain unfrozen at −38.6 °C. It is because the ions brought by the two substances broke the hydrogen bonds between water molecules, making it difficult for them to freeze [42]. In addition, the coordination bond between Li+ and COO− can improve the anti-freezing properties by fixing water molecules. The endothermic peak of PSML-2 hydrogel starts at −34.5 °C, indicating that it has great potential for application in low-temperature environments.

### 2.4. Mechanical Property of Hydrogels

**Figure 3:**
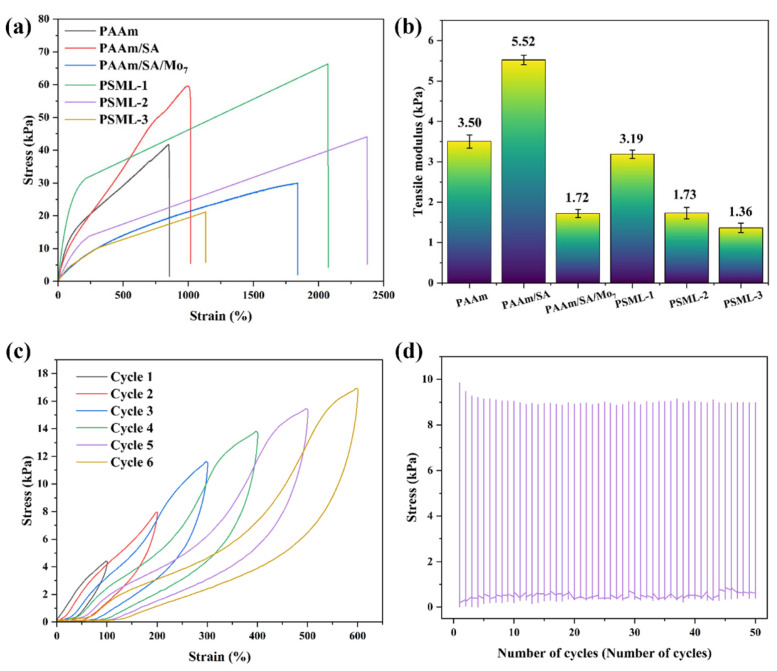
Mechanical properties of hydrogels: (**a**) Tensile curves; (**b**) Tensile modulus; (**c**) Cyclic tensile testing of PSML hydrogel from 100% to 600%; (**d**) Stress-cycle number curve of PSML hydrogel (50 cycles).

The multifunctionality of hydrogels is largely attributed to their exceptional flexibility and extensibility. As shown in Figure 3a,b, the tensile strength of the PAAm hydrogel is 41.49 kPa, the elongation at break is 850%, and the elastic modulus reaches 3.50 kPa. Upon the addition of SA, the tensile toughness of the PAAm/SA hydrogel are significantly enhanced (tensile strength reaches 59.59 kPa, and elongation at break reaches 1016%), and its deformation resistance is also significantly improved (elastic modulus reaches 5.52 kPa). This is attributed to the entanglement between the PAAm and SA molecular chains. The addition of Mo_7_ further enhances the extensibility of the hydrogel, which is attributed to its loose porous structure (as shown in the Figure 1 SEM image comparison). The content of LiCl has a significant effect on the mechanical properties of PSML hydrogel materials. The tensile strength of PSML-1 hydrogel is 66.34 kPa, and the fracture elongation reaches 2073%. Compared with PSML-1, the tensile strength of PSML-2 hydrogel (44.05 kPa) was reduced, but the maximum elongation (2373%) was increased. This result indicates that there is a clear trade-off between strength and ductility in the mechanical properties of the material, with PSML-2 achieving greater deformation capacity at the expense of some strength. The mechanical properties of PSML-3 hydrogel samples were significantly worse, possibly due to excessive LiCl concentration, which led to a decrease in extensibility and brittle fracture. Compared to similar studies [17,30], the tensile strength of PSML-2 hydrogel in this study is similar, but the maximum elongation has increased significantly.

The recoverability and fatigue resistance of PSML hydrogel were further investigated by cyclic strain stretching tests from 100% to 600%. As shown in Figure 3c, PSML hydrogel exhibited hysteresis loops in each cycle, indicating that the hydrogel can enhance its toughness by dissipating energy through the destruction of the hydrogen bond cross-linking network. In addition, the mechanical stability of PSML hydrogel was tested by 50 cycles of stretching at 200% strain. As shown in Figure 3d, the maximum stress during each cycle showed high consistency, indicating that PSML hydrogel has excellent cyclic stretching properties and can meet the stringent requirements of flexible motion sensors and for material cyclic stability.

### 2.5. Photochromic and Fading Properties of Hydrogels

#### 2.5.1. Light Transmission of Hydrogels

**Figure 4:**
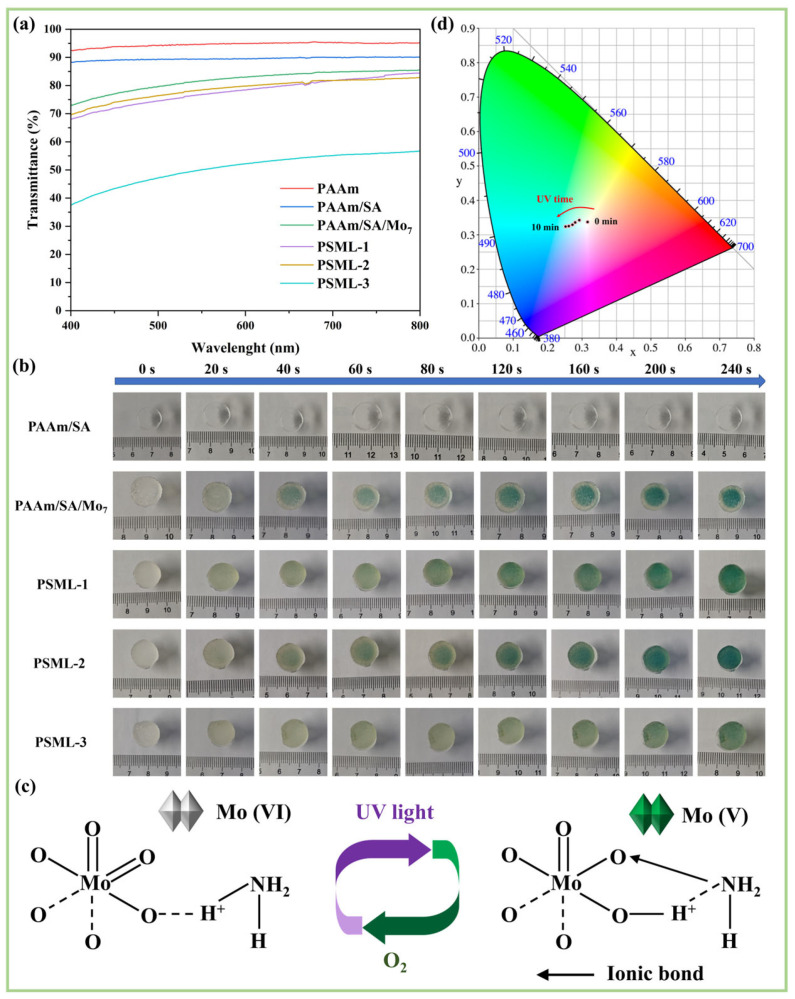
(**a**) Transmittance curve of hydrogels; (**b**) Photos of hydrogels under different illumination time; (**c**) The principle of photochromism of ammonium molybdate; (**d**) CIE coordinates of PSML hydrogel during 10 min UV photochromism.

Figure 4a shows the transmittance curves of the hydrogels. Among them, the transmittance of PAAm is higher than 92%, and SA only slightly reduces the transmittance of PAAm/SA (exceeding 88%). The transmittance of PSML hydrogels decreases due to the addition of Mo_7_ and LiCl. Among them, the transmittance of PSML-3 is below 60%, possibly due to the ‘salting-out’ effect. However, the PSML-2 hydrogel still has a transmittance of over 70%, demonstrating significant application potential in the field of optics.

Therefore, PSML-2 was selected for the subsequent construction of PSML hydrogel due to its optimal comprehensive properties.

#### 2.5.2. The Influence of LiCl Concentration and UV Irradiation Time

Different hydrogel samples were placed under an ultraviolet lamp and exposed to light for 20, 40, 60, 80, 120, 160, 200, and 240 s, respectively, to observe their color-changing behaviour (Figure 4b). The results showed that the PAAm/SA hydrogel did not change color, confirming that Mo_7_ is the photochromic unit of the hydrogel. Notably, the color of the hydrogel first darkened and then lightened with increasing LiCl content at a fixed irradiation time, indicating that LiCl concentration significantly influences the photochromic degree of the hydrogel. Furthermore, the color change time of the PSML-2 hydrogel was less than 20 s, and the coloring efficiency is comparable to that reported in the literature [17,41].

The photochromic process of ammonium molybdate involves reversible redox reactions (Figure 4c). UV light drives the formation of charge transfer complexes (CTC) between ammonium ions and MoO_6_ octahedra, reducing Mo(VI) to Mo(V). The d-orbital electron transition causes coloration (blue at low concentrations and green at high concentrations of Mo_7_). When exposed to air, O_2_ will oxidize the CTC and reverse Mo(V) back to Mo(VI), causing the color to fade [43,44].

As shown in Figure 4d, the CIE color coordinates of the PSML hydrogel gradually shifted towards the blue-green region with increasing UV irradiation time, which further confirmed that the photochromic response of ammonium molybdate mainly occurred in the blue-green spectral range.

#### 2.5.3. Fading Property of PSML Hydrogel

**Figure 5:**
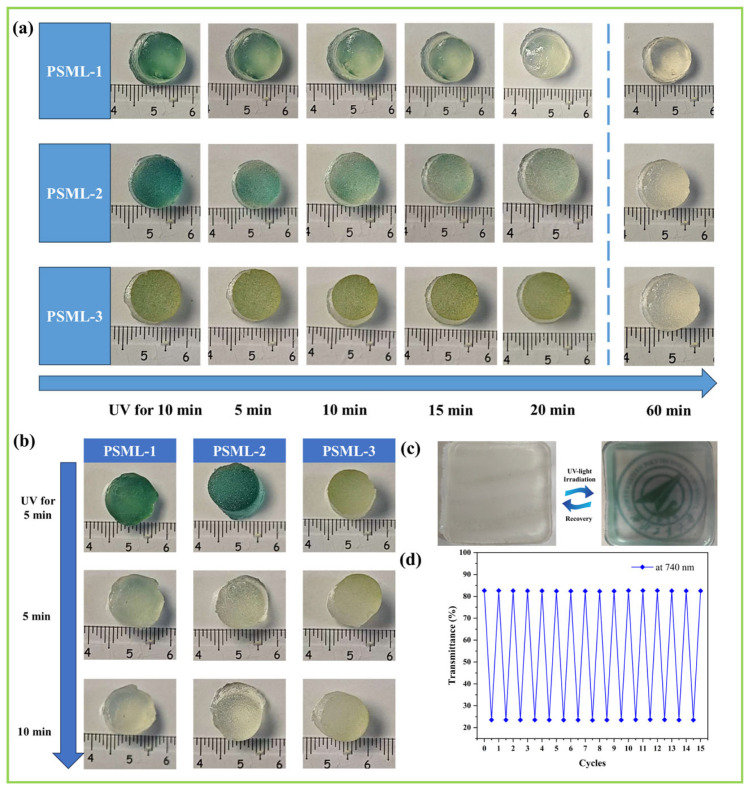
(**a**) The fading process of PSML hydrogels; (**b**) Color change recovery properties of PSML hydrogel under light-shielding conditions; (**c**) Image display function of PSML hydrogel; (**d**) The transmittance changes over 20 color change cycles at 740 nm.

The color-recovery ability of PSML hydrogels with different compositions was also investigated under natural light and in the light-protected environment. As shown in Figure 5a, all three PSML hydrogels slowly recovered to their initial color within 60 min, but the recovery rate slowed as the mass fraction of LiCl in the composition increased. This may be due to the decrease in the solubility of O_2_ in the hydrogel due to the increase in LiCl electrolyte concentration. In the light-protected experiment (Figure 5b), all hydrogels fully recovered within 10 min, indicating that a dark environment is conducive to the recovery of PSML hydrogels.

As shown in Figure 5c, the repeatable color-changing and fading properties of the hydrogel make it suitable for image display applications. To assess the long-term stability of the hydrogel’s reversible color-changing ability, the transmittance at 740 nm was monitored using ultraviolet–visible spectroscopy (UV–Vis) during 20 color-changing cycles (Figure 5d). The results showed that the transmittance of the sample remained unchanged after 20 cycles, confirming the hydrogel’s excellent color change cyclic stability, which may facilitate its application in information encryption.

##### 2.6. 3D Printing of the PSML Hydrogels

**Figure 6:**
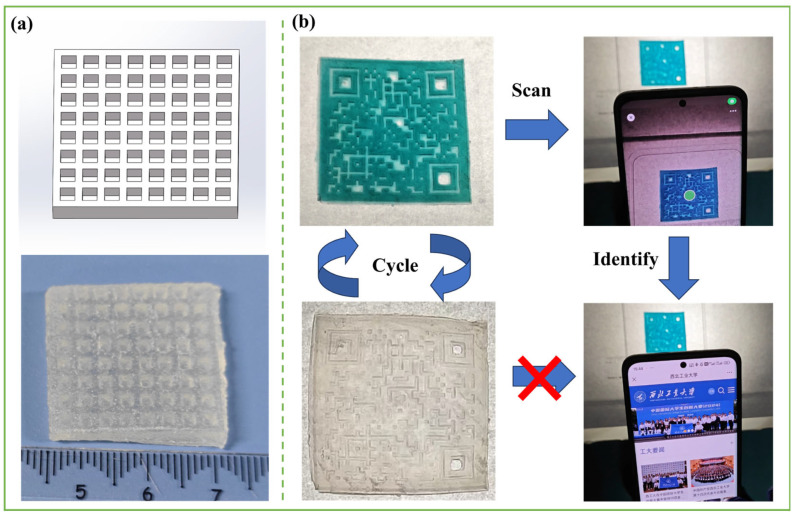
(**a**) 3D printed lattice structures; (**b**) 3D-printed hydrogel with QR code.

**Figure A1:**
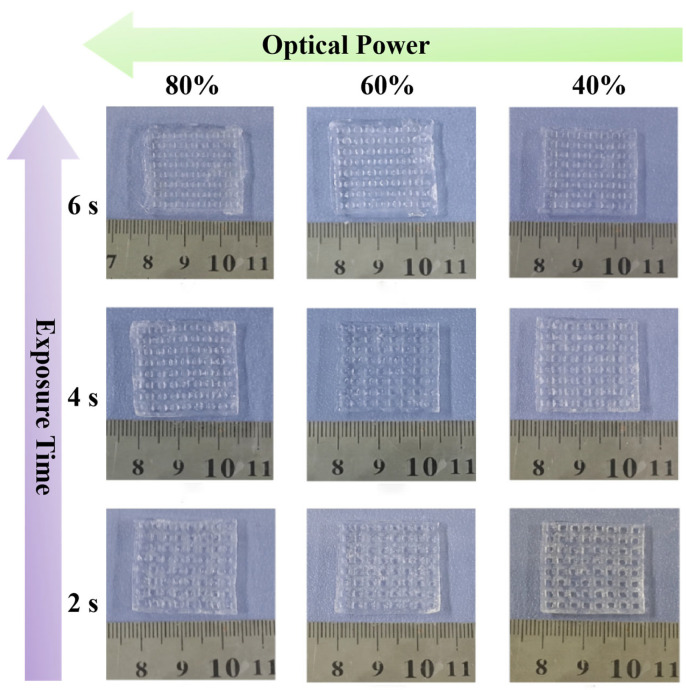
3D printing effects of PSML hydrogel under different printing parameters.

The PSML-2 hydrogel precursor solution was selected for 3D printing testing to determine the optimal printing parameters (Figure A1). As shown in Figure 6a, a three-dimensional hydrogel with an 8 × 8 grid structure (side length 32 mm) was successfully fabricated using the DLP printing process, which fully demonstrates the excellent printability of PSML hydrogel and the high-resolution characteristics of this process. Notably, a hydrogel structure with a QR code pattern was further printed based on the precise molding capabilities of the photopolymerization technology (Figure 6b). Due to the high transparency of PSML hydrogels, the QR code cannot be recognized by a smartphone under normal conditions. However, the QR code information can be easily recognized by a smartphone thanks to the photochromic properties of Mo7 after UV light exposure (Video S1). The results confirm the significant application potential of such photochromic hydrogels in the field of information encryption.

### 2.7. Electronic Skin

**Figure 7:**
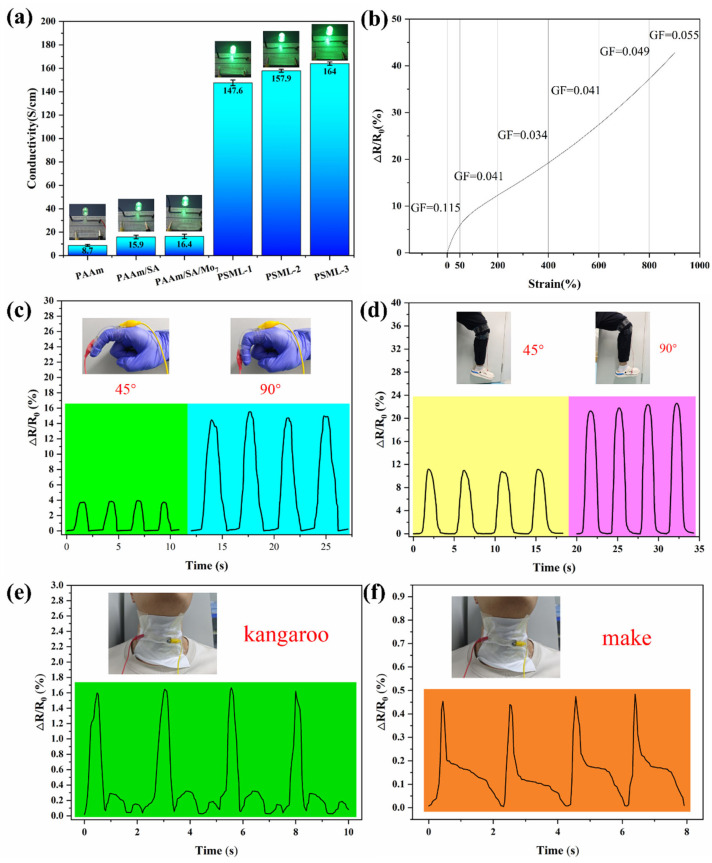
(**a**) The conductivity of hydrogels; (**b**) GF value of PSML hydrogel sensor during tension under 0 to 500 % strain; Sensing signals of (**c**) finger bending, (**d**) leg lifting, (**e**) speaking “kangaroo”, (**f**) speaking “make”.

Figure 7a shows the conductivity of different hydrogels and the resulting changes in LED brightness under a 3V circuit connection. Clearly, the introduction of LiCl significantly enhances the conductivity of the PSML hydrogel. Among these, the PSML-3 hydrogel exhibits a conductivity as high as 164 S/cm, which is attributed to the directed migration of Li+ ions under the influence of an electric field. The conductivity of the PSML hydrogels is comparable to that of previously reported ionic hydrogels [45,46,47].

The gauge factor (GF) is a key indicator of a material’s strain sensitivity. As shown in Figure 7b, the relative change in resistance (ΔR/R_0_) curve of PSML hydrogel within the 0–900% strain range can be divided into three characteristic regions. In the 0–50% strain region, the GF value reaches 0.115, confirming its high strain sensitivity and suitability for strain sensors. In the 50–400% strain region, the GF value sharply decreases to 0.034, indicating that the resistive response significantly weakens under high strain conditions. In the 400–900% strain region, the GF value gradually increases to 0.055, reflecting that the material’s sensitivity improves as strain increases.

With its high strain sensitivity, the PSML hydrogel sensor can accurately monitor human movements. When the finger is bent at 90°, the relative resistance change of the PSML hydrogel strain sensor is >14% (Figure 7c). The hydrogel sensor also exhibits excellent repeatability in its resistance response to movements such as leg lifting (Figure 7d), making it suitable for human motion recognition. Furthermore, the hydrogel sensor exhibits unique strain response signal characteristics for human voice vibrations (Figure 7e,f). In summary, hydrogels can achieve multi-scale recognition from large-deformation movements to micro-vibration voice signals, demonstrating significant application potential in fields such as human–machine interaction, health monitoring, and electronic skin.

## 3. Conclusions

In summary, we successfully synthesized a series of photochromic hydrogels based on the polyoxometalate Mo_7_ with ultra-high electrical conductivity using the one-pot method and 3D printing. Among them, the PSML-2 hydrogel exhibited the fastest UV response speed (<20 s) with an electrical conductivity as high as 164 S/cm. Notably, the addition of LiCl not only enhances the hydrogel’s freeze resistance but also improves its photochromic properties to some extent. Benefiting from the synergistic effect of the PAAm/SA dual network and the photosensitive material Mo_7_, this hydrogel exhibits reversible photochromic properties, excellent stretchability (>2300%), printability, and high strain sensitivity (GF = 0.115), laying the foundation for its application in flexible strain sensors and information encryption devices. However, the system still faces numerous challenges in practical applications: the environmental stability of hydrogels (such as water evaporation and long-term chemical stability when exposed to air) is critical to their service life, especially in dry or high-temperature environments where performance maintenance poses challenges; furthermore, although the introduction of LiCl has improved freeze resistance, conductivity and mechanical properties under extreme low-temperature conditions still require further optimization. We believe this research will provide insights for the design of smart electronic skin, advanced display materials, and photoelectric dual-response sensors, and guide the development of three-dimensional rapid manufacturing and information encryption functions for Mo_7_-based smart electronic skin.

## 4. Experiments

### 4.1. Materials

Ammonium molybdate tetrahydrate (Mo_7_) (AR, 99%), N,N′-methylenebisacrylamide (MBA, AR), lithium chloride (LiCl, AR, 99%), ammonium persulfate (APS, AR, 98.5%), and acrylamide (AM) (AR, 99.0%) were bought from Macklin Biochemical Technology Co., Ltd. (Shanghai, China). Sodium alginate (SA) (CP, 2000 mPa·s) was purchased from Qiansheng Biotechnology Co., Ltd. (Hefei, China). Lithium phenyl (2,4,6-trimethylbenzoyl) phosphate (LAP, 99%) was purchased from Shanghai Shifeng Biotechnology Co., Ltd. (Shanghai, China). Deionized (DI) water was homemade in the lab.

### 4.2. Preparation of the PSML Hydrogels

**Figure 8:**
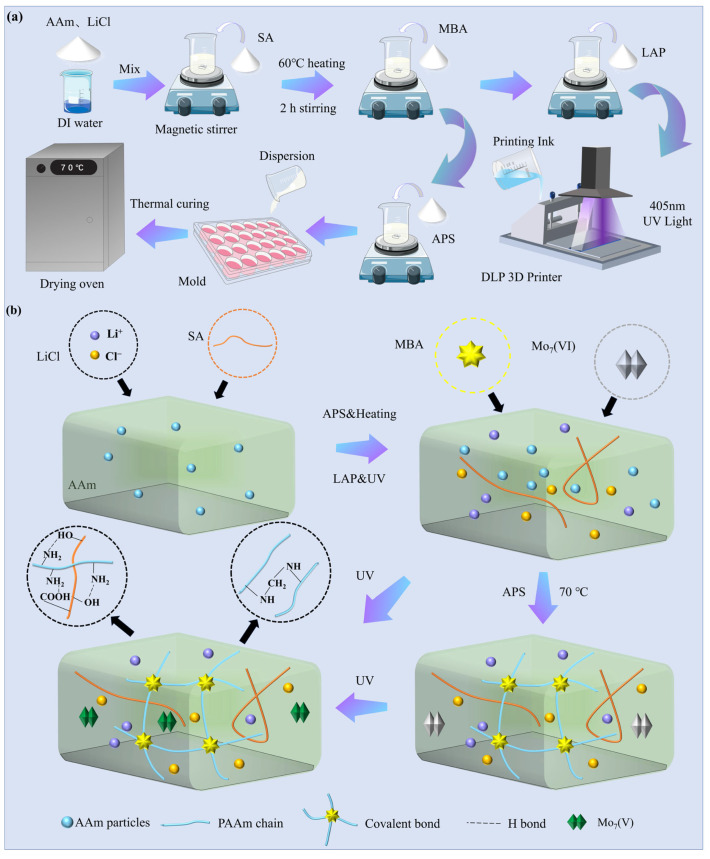
(**a**) Preparation process of PSML hydrogel; (**b**) Experimental mechanism diagram.

The PAAm/SA/Mo_7_/LiCl (PSML) hydrogel prepolymerization solution can be directly produced by a one-step method (Figure 8a). Firstly, AM, LiCl, and Mo_7_ were dissolved in deionized water at 60 °C to form a clear, transparent, and homogeneous solution, and then SA was added into the solution and stirred at high speed, and the stirring was stopped when there was no obvious agglomeration of SA, and a total of 60 g of well-dissolved and uniformly dispersed hydrogel prepolymerization solution was obtained.

PSML hydrogels can be prepared by thermal curing. In the preparation of heat-cured PSML hydrogels, MBA (6 mg) and APS (45 mg) were added to the prepolymer solution, stirred briefly, and then thermally polymerized at 70 °C for 1 h. This method yields PSML hydrogels with moderate crosslink density and uniform network structure. The composition details of all hydrogel components are provided in Table A1.

### 4.3. 3D Printing of Hydrogels

First, 56.26 mg of MBA and 0.12 g of LAP (0.2% wt) were dissolved in the hydrogel precursor solution and stirred at 60 °C for 1 h to obtain 60 mL of PSML ink. The PSML ink must be protected from light prior to 3D printing due to the high sensitivity of the photoinitiator to light. 3D-structured PSML hydrogel was fabricated using a DLP photopolymerization 3D printer (J2-D96P-CERAMICS, JUNJING Technology, Foshan, China). The main parameters of 3D printing mainly include optical power (60%), layer thickness (100 μm), and exposure time (4 s). Figure 8b shows the reaction mechanism of hydrogel preparation.

### 4.4. Characterization

#### 4.4.1. Constituent Analysis

The FTIR spectra of PSML hydrogel samples (500–4000 cm^−1^) were measured using a diamond ATR attachment on a BRUKER ALPHA II (ALPHA II, BRUKER, Karlsruhe, Germany) spectrometer.

#### 4.4.2. Morphological Characterization

The hydrogel material was dehydrated and freeze-dried using a vacuum freeze-dryer (TF-FD-27, Tianfeng, Shanghai, China) to prepare the sample. The freeze-dried hydrogel sample was then broken in half and fixed on the sample stage with the cross-section facing upwards for easy observation. To ensure that the sample could be clearly observed under a tungsten filament lamp electron microscope (Tescan Vega4, TESCAN, Brno, Czech Republic), the conductivity of the sample surface was enhanced by spraying it with gold.

#### 4.4.3. Mechanical Performance

A universal testing machine (CTM2500, Xieqiang Instruments, Shanghai, China) was used to measure the stress–strain curves of hydrogels with different compositions by performing tensile tests on rectangular ionic hydrogels (L = 50 mm, W = 12 mm, H = 3 mm) at a speed of 50 mm/min. At a tensile rate of 50 mm/min and a maximum strain of 200%, the rectangular hydrogel sample was subjected to 50 cyclic tensile tests, yielding the stress–strain curves under cyclic tensile conditions. The value of the tensile modulus of elasticity (E) corresponds to the slope of the stress–strain curve when the strain is from 300% to 800%.

L, W and H represent the length, width, and height of the sample, respectively. The meanings of the three letters provided below are the same.

#### 4.4.4. Differential Scanning Calorimetry (DSC) Measurement

To investigate the freeze resistance of hydrogels, differential scanning calorimetry (DSC) was used to analyze changes in heat flow during the cooling process of hydrogel materials. In the experiment, the hydrogel samples were tested by cooling them from 20 °C to −120 °C at a rate of 5 °C/min and observing their crystallization peaks.

#### 4.4.5. Transmittance Measurement

In this study, the transmittance of hydrogels with different compositions was measured using a UV spectrophotometer (T6 New Century, PERSEE, Beijing, China). In this experiment, hydrogels (L = W = 45 mm, H = 1.5 mm) with different compositions were placed in a fixture and inserted into the UV spectrophotometer for measurement. The transmittance of the hydrogel materials with different compositions was obtained through spectral scanning under illumination at wavelengths ranging from 400 nm to 800 nm.

#### 4.4.6. Photochromic Exhibition of Hydrogels

The experiment used ultraviolet light with a wavelength of 365 nm to irradiate PSML hydrogel at a height of 25 mm, and the color change process of each sample group was photographed and recorded. The source of UV irradiation comes from an LED UV lamp (35 W, Tiandou Lighting, Zhongshan, China). Hydrogel samples with different compositions were prepared into cylindrical samples (d = 10 mm, H = 5 mm). In the light-protected recovery test, the sample was first exposed to a 365 nm ultraviolet lamp for 5 min then stored in the dark. It was taken out every 5 min and its color change was observed under natural light. The photochromic spectrum of PSML hydrogel was measured using a spectrophotometer (HP 350, Hangzhou Shuangse, Hangzhou, China).

*d* represents the diameter of the sample; the meaning of the letter provided below is the same.

#### 4.4.7. Conductive Properties

To visually demonstrate the electrical conductivity(*σ*) of PSML hydrogel samples (L = 40 mm, W = H = 2 mm) and the effect of strain on their electrical conductivity, the electrical conductivity (*σ*) of PSML hydrogel under different compositions can be further calculated based on the specific cross-sectional parameters of the samples. The conductivity of each hydrogel was calculated as the average value of 5 samples. The calculation formula is as follows:

$$\sigma =\frac{l}{Rs}$$

where *l*, *s*, and *R* are the length, cross-sectional area, and resistance of the PSML hydrogel, respectively.

In addition, the gage factor (*GF*) used to characterize the strain sensitivity of the hydrogel sensor was calculated by the following formula:

$$GF=\frac{(R-R_{0})/R_{0}}{\varepsilon }$$

where *R*_0_ and *R* are the initial and post-deformation resistances, respectively, and *ε* is the strain of the PSML hydrogel.

## Data Availability

The original contributions presented in this study are included in the article/Supplementary Material. Further inquiries can be directed to the corresponding author(s).

## Supplementary Materials

The following supporting information can be downloaded at: https://www.mdpi.com/article/10.3390/gels11090703/s1, Video S1: The process of 3D-printed PSML hydrogel QR code being recognized by a mobile phone after color change.

## Appendix A

**Table A1:** Design of hydrogel prepolymerization solution components.

Hydrogels	Aam (g)	SA (g)	Mo_7_ (g)	LiCl (g)	H_2_O (g)	MBA (mg)	APS (mg)	LAP (g)
PAAm	9	0	0	0	50.95	6	45	0
PAAm/SA	9	2	0	0	48.95	6	45	0
PAAm/SA/Mo_7_	9	2	0.7	0	48.25	6	45	0
PSML-1	9	2	0.7	3	45.25	6	45	0
PSML-2	9	2	0.7	6	42.25	6	45	0
PSML-3	9	2	0.7	9	39.25	6	45	0
PSML ink	9	2	0.7	6	42.12	56.26	0	0.12
